# Adaptations and staff experiences in delivering parenting programmes and other family support services in three community-based organisations in Cape Town, South Africa during the COVID pandemic

**DOI:** 10.1080/17441692.2022.2129725

**Published:** 2022-11-07

**Authors:** Yulia Shenderovich, Hlengiwe Sacolo-Gwebu, Zuyi Fang, Jamie Lachman, Lucie Cluver, Catherine Ward

**Affiliations:** a Wolfson Centre for Young People's Mental Health, Cardiff, UK; b Centre for the Development and Evaluation of Complex Interventions for Public Health Improvement (DECIPHer), School of Social Sciences, Cardiff, UK; c Department of Social Policy and Intervention, Centre for Evidence-Based Intervention, University of Oxford, Oxford, UK; d Department of Psychology, University of Cape Town, Cape Town, South Africa; e School of Social Development and Public Policy, Beijing Normal University, Beijing, People's Republic of China; f MRC/CSO Social and Public Health Sciences Unit, University of Glasgow, Glasgow, UK; g Department of Psychiatry and Mental Health, Groote Schuur Hospital Observatory, University of Cape Town, Cape Town, South Africa

**Keywords:** Families, children, parenting, South Africa, COVID-19, pandemic

## Abstract

We explore how organisations working on parenting programmes and other types of family support and violence prevention in low-resource settings experienced the pandemic. In August 2020–May 2021, we interviewed (1) staff from three community-based organisations delivering evidence-informed parenting interventions and other psychosocial services for families in Cape Town, South Africa, (2) staff from a parenting programme training organisation and (3) staff from two international organisations supporting psychosocial services in South Africa. Interviews (22) were thematically analysed, with findings in three areas. First, respondents noted changes in the context, including the job losses, food insecurity, and stress experienced by local communities, and reductions in organisational funding. Second, we found that in response to these context changes, the organisations shifted their focus to food provision and COVID prevention. Parenting and psychosocial programmes were adapted – e.g. by changing the physical delivery settings, reducing group sizes, and taking up digital and phone implementation. Participants reported improved perceptions of remote delivery as a feasible approach for working with families – but internet and phone access remained challenging. Third, the pandemic brought new responsibilities for staff, and both the challenges of working from home and the health risks of in-person work.

## Introduction

Violence against children has become recognised as a key public health issue. Parenting programmes and programmes to support food security are among the research-informed recommended strategies for reducing the risk of violence against children and harsh parenting (Kohli et al., 2021; World Health Organization, 2016). The pandemic has created new pressures on children and families, such as increased poverty and stress, accompanied by decreased access to social support and to services, such as childcare, thus elevating risks of violence against children and harsh parenting (Bhatia et al., 2020, 2021; Bourgault et al., 2021; Fabbri et al., 2021; Katz et al., 2021). Increases in calls to child helplines were reported in a number of countries (Cappa & Jijon, 2021; Petrowski et al., 2021) – for example, Childline South Africa, a telephone hotline for children to report maltreatment, reported a 400% increase in calls during the lockdown period in 2020 (Katz et al., 2021).

Several studies have documented lessons learned from adapting services, such as parenting programmes, during the pandemic (Cook et al., 2021; Fogler et al., 2020; Garcia et al., 2021; Riegler et al., 2020). Much of this research has focused on programmes delivered within research projects. Therefore, there is a gap in understanding how organisations working with families in routine service delivery in low-resource settings experienced and adapted to the impacts of the pandemic. The aim of this paper is to explore how organisations and their staff working with families, have adapted. We focus on the experiences of three community-based organisations in Cape Town, South Africa, complemented by the wider perspectives of staff from funding and training organisations in South Africa.

## Materials and methods

### Study design

This research was conducted as part of a project studying the implementation of Parenting for Lifelong Health (PLH), a suite of evidence-informed parenting programmes designed to reduce violence against children and promote positive parenting, including PLH for Young Children (2–9 years), and PLH for Parents and Teens (10–17 years) (for protocol and more information see Shenderovich et al., 2020, 2021). The PLH for Young Children programme is delivered to caregivers, while PLH for Parents and Teens includes both caregivers and adolescents. Both programmes are group-based and are delivered by two group leaders (‘facilitators’), working with a group of families over a course of weekly sessions for 2–3 months. The facilitators receive ongoing supervision and support on group process from more experienced colleagues (‘coaches’). The work of facilitators and coaches, and the overall logistics of the programme are usually overseen by a coordinator or a manager. Both programmes have been tested in randomised controlled trials with positive impacts on reducing child maltreatment and improving parenting behaviours as well as other child and family outcomes (Cluver et al., 2018; Ward et al., 2020).

### Recruitment

We recruited three community-based non-governmental organisations (NGOs) delivering PLH programmes and other services for families in disadvantaged areas of Cape Town. Based on the principle of maximum variation (Creswell & Poth, 2016), we selected NGOs that represent a range of organisational sizes and target populations to reflect different perspectives (see Table 1 for organisational profiles).

**Table 1.** Overview of the activities of the participating community-based organisations.

	Organisation A	Organisation B	Organisation C
Founded	1964 informally, 1992 formally	1989	2018
Staff members	123	9	4
Volunteers	1	1	2
Main areas of work	Senior citizen clubs, support for elderly, educares for young children, after-school clubs for teens, psychosocial support team, health programmes (e.g. vision screening), parenting programme	Support for elderly, parenting and fatherhood programmes, sustainable livelihoods, community feeding programme	Family support, education support, support for school dropouts, self-defence classes, skills development, psychosocial support, including a parenting programme, addressing substance use
Main areas of work during 2020–2021	Food distribution, counselling, referrals to social workers for assistance with various social problems including domestic violence, lack of South African IDs	Food distribution to the community	Food distribution and help with government poverty-relief grant applications
PLH programmes	PLH for Young Children and PLH for Parents and Teens	PLH for Parents and Teens	PLH for Parents and Teens
PLH delivery start	2018	2020	2020
Families in PLH	∼160 families	∼20 families	∼8 families

Next, we employed snowball sampling (Creswell & Poth, 2016; Parker et al., 2019). After obtaining organisational consent, we asked contact persons in each organisation to disseminate information about the study, provided by the researchers, to all staff involved with PLH delivery, emphasising that participation was voluntary. This included programme facilitators (those who deliver services to families, both paid employees and volunteers), coaches (those who provide supportive supervision to facilitators), and coordinators (those who coordinate programme delivery). The contact person in each organisation shared only the details of the individuals who were interested to participate in the research with the research team, and participants were then guided through individual informed consent by trained researchers.

To provide a wider perspective of the impact of COVID-19 on organisations working with families, we also recruited and interviewed staff from Clowns Without Borders South Africa, an NGO based in Cape Town that provides training and technical support for organisations implementing PLH in South Africa and other countries. We also recruited staff working for two international funding agencies that support parenting programmes and other family support services in South Africa. These individuals were invited and recruited directly rather than via their organisations.

### Data collection tools and procedures

To provide consistency across interviews, as well as flexibility to explore emerging topics, we used semi-structured interviews (Bowling, 2014; Parker et al., 2019). The interview topic guides (available as supplementary materials and on project page: https://osf.io/9ydjz/) were designed prior to the data collection and piloted during the initial interviews by YS and HS. The piloting process suggested that no major modifications were needed, although some prompts were amended. The questions about experiences during the pandemic encompassed the period from the arrival of the pandemic in South Africa around March 2020 until the interviews took place from August 2020 to May 2021 – roughly, the first year of the pandemic for South Africa.

Interviews were conducted by phone or online. Each participant took part in one interview, sometimes within multiple consecutive online or phone conversations due to lost connection. The interviews were between 39 and 107 minutes long (average: 78 minutes). HS and YS conducted the interviews in English, isiXhosa and Afrikaans, with an interpreter providing support for interviews in Afrikaans. Audio recordings of the interviews were transcribed, and, for the isiXhosa and Afrikaans interviews, translated into English prior to analysis. The transcripts were shared with participants for checking.

### Participant characteristics

Relevant data was drawn from interviews with 22 participants: 14 parenting programme facilitators and coaches, three coordinators, three staff from the parenting programme training organisation, and two staff from international organisations in South Africa. In the three NGOs, nearly all programme staff who had worked with PLH were able to participate in the study. Of the 22 participants, 20 were female and two were male. We did not collect data on age and other demographic characteristics as this was deemed not essential to the study focus.

### Data analysis

Interview data were analysed using the thematic framework approach (Gale et al., 2013; Heath et al., 2012). First, YS, HS and ZF became familiar with the data by listening to the interviews and reading interview transcripts and notes multiple times. Second, themes related to the pandemic experiences were identified by YS, HS and ZF, and constructed into an initial coding framework. Third, using this framework, 10% of the interviews were independently coded by YS and ZF. Any disagreements between the coders were resolved through discussion, leading to further refinement of the coding scheme. Fourth, we finalised the coding framework and applied it to all transcripts. The authors met regularly to discuss the themes and compare across individual and organisational experiences of the pandemic. To facilitate the analysis, we created a matrix in Microsoft Excel, where columns represented the themes, and rows represented participants, with summaries of their responses and quotes relevant to each theme. During coding, the transcripts were labelled based on the participant role – interviews with parenting programme facilitators and coaches were labelled F1, etc., interviews with coordinators and managers as M1, etc., interviews with staff at the training organisation for the PLH parenting programme as T1, etc., and with international organisation staff as S1, etc.

In this paper, we focus on the experiences related to the pandemic, during the period from the arrival of the pandemic in South Africa around March 2020 up until the interview. Other themes, related to the implementation and sustainability of PLH were analysed separately and will be presented elsewhere (Sacolo-Gwebu, In submission).

The interpretation of the data by the research team was checked with the study participants. We held validation meetings in the participating NGOs, where the findings were presented by the researchers, and the participants were encouraged to provide feedback on the findings. The participants indicated that the results were consistent with their experiences, and provided additional details and clarifications, which were incorporated into this analysis. All participants received a draft of this paper for comments and were encouraged to provide feedback. We report this manuscript using the COREQ checklist (Tong et al., 2007).

### Reflexivity

HS managed the introduction of the study and the interview scheduling. YS and HS conducted the interviews. Both are female researchers with PhDs in social and health research. YS previously implemented and conducted research on parenting programmes, HS had experience in community-based projects. ZF is a female researcher who was conducting her PhD on programmes for parents of children with autism. YS is based in the UK, ZF in UK and China, and HS in South Africa. YS, CW, LC, and JML have worked on previous studies together. CW and several members of the research team have previously collaborated with organisation A and some of the stakeholders interviewed. JML, LC and CW work on the PLH programme development. JML is the founder and previous director of Clowns Without Borders South Africa and is a master trainer of PLH programmes. To mitigate social desirability bias, the participants were informed at the start of every interview and throughout, where relevant, that the research team are interested in understanding implementation experiences and are not in evaluating the work of the organisations, and that all opinions were welcome.

### Ethics

The study has received ethical approval from the Universities of Cape Town (PSY2017-040) and Oxford (SPICUREC1a__20_015).

## Results

**Figure 1.**
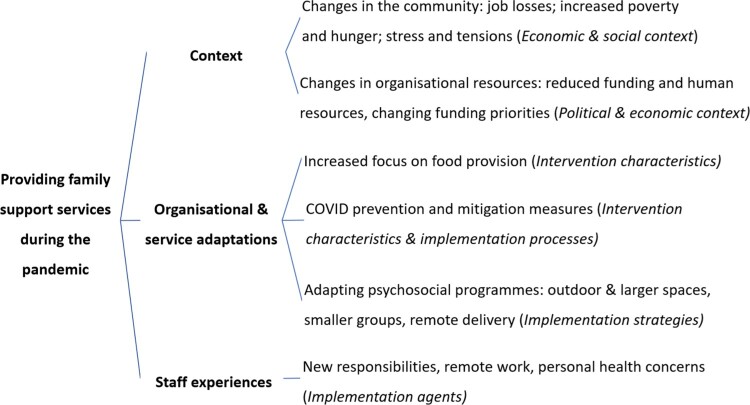
Overview of the themes (dimensions of the CICI framework in italics).

Three groups of themes emerged from the data: (1) changes in the context of the local communities and community-based organisations, (2) adaptations of the services that organisations provide and (3) staff experiences during the pandemic (see Figure 1 for an overview). Although we did not ask specifically about how the communities where the participants worked were affected by COVID, participants shared their reflections on the perceived impacts of COVID, as context for how their work has been affected by the pandemic and adapted to it. Given the importance of context in our findings, we drew on concepts from the Context and Implementation of Complex Interventions framework (CICI; Pfadenhauer et al., 2017), which was developed through literature reviews and iterative application.

### Changes in the context

#### Changes in the community

In addition to the immediate health risks and impacts of the pandemic, the most highlighted aspect of how the disadvantaged communities in Cape Town had been affected by COVID, in participants’ perception, were the economic and social changes – increased poverty and job losses, which increased hunger and food insecurity. Participants also linked this to increased stress and tensions in families:

Because most of the people didn't go to work. Most of the people lose their jobs. So, you didn't have food to eat, and the children were also affected, you know. (M2)

… there is frustration because there is less food to go around everyone and everyone is always panicking (F8)

Respondents reported that challenges also arose from families spending a lot of time together at home, often in overcrowded conditions, due to school closures and restrictions on movements.

#### Changes in organisational resources

Participants from all three NGOs in Cape Town talked about reduced funding and challenges with raising funds during the pandemic. Interviewees from organisations B and C reported reductions in staff related to the pandemic – organisation B because of redundancies due to reduced funding, and organisation C due to its volunteers needing to find other, paid work. Participants also talked about changes in raising funds for their work due to the pandemic: ‘You know, we had to raise funds, especially for group sessions when the parents come, so COVID sort of escalated that problem. We could not inquire; we could not raise funds; we could not meet for fundraising’ (M3).

Similarly, a respondent working with an international organisation that funds and provides technical support for services for families across South Africa observed a shift in the priorities of local and national governments away from family support programmes towards COVID-19 response efforts, reallocating funding to respond to the pandemic:

[Many provincial governments in South Africa] had indicated that the budget that they had, had been reprioritised on COVID response. So any services […] would be focused on […] families who've been affected by COVID. So, a lot of our normal programming actually came to a halt with government. (S1)

### Organisational and service adaptations

#### Increased focus on food provision

Each of the three community-based organisations included in this study provides multiple services for families, such as parenting programmes and after-school clubs, as listed in Table 1. Food provision became a key organisational activity during the first year of the pandemic. Each of the three NGOs has been offering some type of food support, such as food parcels delivered directly to households or soup kitchens where food can be picked up, whereas only one NGO had offered food support before the pandemic.

So, we never stopped delivering food parcels because the other people, they lost their jobs, and they not working anymore. So we will just bring them something to put on their tables. Something to eat. (F2)

So, we were allowed [to run a feeding site during COVID-related restrictions on gathering] because providing food was under emergency services. You understand, [the other programmes were paused], but the feeding had to continue. (M1)

It became pretty [attractive] again for people to fund food [support] during COVID [compared to prior to the pandemic] (validation meeting in organisation A)

#### COVID prevention and mitigation measures

Organisations A and B also adopted new activities related to COVID prevention and mitigation, such as making face masks, increasing awareness through pamphlets and conversations, and providing advice about COVID:

There would be pamphlets, and the people who deliver the food would try to explain at least for 5 minutes, trying to explain and making sure the family do understand how to do the social distancing, hand washing and all that. (F8)

The biggest challenge is, when you find out this person, or this house, was a positive in COVID, and then you find out the space is too small. […] Now you have to tell them to be cautious, and to stay in one place, not go around the house, because it will infect the others in the household. (F2)

Participants also talked about the safety measures that were implemented for protecting staff and families involved in their work. In addition to adapting the physical spaces for group meetings, as discussed next, safety measures included keeping distance from each other, wearing masks, washing and sanitising hands during activities, and keeping contact details to notify people who were exposed during psychosocial or food programmes, in case of an infection.

#### Physical and digital adaptations of parenting and other psychosocial programmes

Many participants commented on how COVID risks and restrictions, and the related uncertainty, reduced their ability to meet families in groups and to visit households. This led to a *pause in delivery* of in-person family support programmes, such as the Parenting for Lifelong Health programmes and the after-school clubs, in the second half of 2020 and early 2021. As one facilitator put it, ‘When the lockdown began, we stopped meeting the families and children. It got too difficult’. (F7).

Lack of outdoor and large indoor spaces to work with groups of children and families was a barrier to delivering group-based services, particularly during the closures of public spaces such as playgrounds in South Africa. In 2021, all three NGOs started to engage with families again in person, shifting to working with smaller groups of families and using spacious indoor or outdoor venues. ‘Either we have bigger spaces or reduce the number of people per group session so that we can do social distancing’, one of the programme facilitators shared (F5). Another strategy to reduce COVID risk, explored by one of the international funding organisations in South Africa, was to shorten programme length, thereby reducing the number of in-person meetings and potential COVID transmission.

Organisation A also adopted two digital adaptations of the PLH programme designed by the PLH developers during the pandemic: a WhatsApp-based group led by a facilitator and a series of one-sided informational sheets that distilled core components of PLH into tips. The tip sheets were used digitally and as printouts, for instance, distributed with food parcels. The respondents working for funding agencies gave examples of the use of tip sheets in multiple parts of South Africa. The capacity building organisation Clowns Without Borders South Africa also started to provide Zoom-based facilitator training. Overall, the participants involved in funding and training in parenting programmes talked about shifting perceptions in favour of digital staff training and digital or phone delivery of parenting programmes:

You'll find that before Corona people never thought that the remote support or virtual meetings are possible. So, they say, no, we have Internet problems, we cannot do it, we need to face-to-face. […] And now we are looking into a digital platform, because through Corona our experience is that we need to change the way you think. (T1)

We've allowed our implementing partners to actually invest a little bit in exploring digitisation, something that two years ago we were not very interested in supporting. Last year we have gone full steam ahead and accepted that, you know, we gonna need a learning management platform. (S2)

A key challenge reported by frontline staff as well as other stakeholders in respect to delivering remote programmes was that many families and programme facilitators did not have a smartphone or sufficient phone credit and mobile data to allow them to participate fully in chat groups on WhatsApp and similar.

… when you come up with digital strategies – are parents even gonna have the devices that are needed for them to access such? (T2)

Only a few parents have phones … I realised when I was opening the group that some do not have WhatsApp … [But] most have access to WhatsApp through other members of the family. (F3)

Several frontline staff members also shared concerns about how online and phone platforms limited their ability to get to know the family well, establish strong relationships, and support families who were not engaging.

It is hard now, during COVID, because there is a lot [of people who] we cannot reach. For example, with recruiting, it is difficult now to go to families as we would like to see them in person. When you are with the person, it is much better than talking to them over the phone. (F6)

Participants from the smaller NGOs (B and C) also reported difficulties compiling up-to-date contact details for families, which both organisations were still collecting at the time of the interviews. The respondents suggested that addressing these barriers would be crucial in working with families remotely: ‘The other problem is that we did not have a complete list of the parents, their numbers and where each one lives. But now we have seen, and we are preparing a good list’ (M1).

### Frontline staff experiences

Staff members found themselves with changed or new job responsibilities due to the pandemic. New tasks posed challenges – ‘we were told to make masks. Sewing is not our thing; we were never taught that’ (F3). There was also a mobilisation of effort – for example, a programme coordinator said ‘ … no one was saying this isn’t my role. We all went into feeding, helped into feeding’ (M2).

Generally, the participants saw phone and online communication during the pandemic as helpful to facilitate their work: ‘It was not that hard to continue working and what we would do when we had a meeting was to use Zoom and work through the phone’ (F4). At the same time, there were challenges related to lack of mobile data and poor connection: ‘we [facilitators] often run out of airtime and have things that we should have done with it’ (F6); This was particularly pronounced for community volunteers: ‘we, as members of community organisations, how do we all connect on Zoom? Not everyone has got Zoom, and not everyone has got internet’ (M1). Participants suggested that providing mobile devices and sufficient phone and internet credit was key for enabling remote work.

Another challenge was the reduced informal communication with colleagues: ‘staying at home without meeting at the office has difficulties because at the office it is easy to have a meeting and advise each other’, one facilitator commented (F3). Several participants also noted the stress and tiredness related to working remotely, with long periods in front of a computer or phone, and without built-in breaks and social interactions:

It's exhausting because you don't say I work at 8:00 and being out at 17:00. We work even from as early as 5:00 o'clock until 22:00 o'clock in the evening, preparing … It's not like going and doing the work. (M2)

At the same time, in-person work with families brought its own challenges due to personal health concerns*.* Several participants shared concerns about their own health or health of family members, related to age and chronic illness, putting them at a higher risk from a COVID infection.

I am typically the first person to ask to be excused from work, since COVID-19 is very infectious to people with underlying conditions. This was frustrating because I did not know what to expect after that, but I had to come back to work because I am in need of a job – but it is risky (F4).

## Discussion

Our study examined how organisations working with families in Cape Town, South Africa, managed the pandemic and its consequences. We focused on three community-based organisations and included wider perspectives from training and funding organisations. The comments from the different groups of interviewees were generally consistent and identified three main areas of influence of the COVID pandemic – changes in the community, such as increased poverty and the closure of schools, leading to mounting pressures on families, while organisational resources often became more limited; adaptations of the formats for existing psychosocial programmes and an increased focus on food provision; and new staff responsibilities and work patterns.

Our findings highlight the impact of increased poverty related to the COVID pandemic as a key challenge for families. Other recent research in South Africa, both quantitative and qualitative, also found increases in food insecurity, unemployment, and poverty, particularly among the poorest communities and individuals (Gittings et al., 2021; Haffejee & Levine, 2020; Jain et al., 2020; Schotte & Zizzamia, 2021). This context shaped the services of organisations working with families. The community-based organisations included in our study focused on food provision and adopted public health duties, such as dissemination of face masks and information about COVID.

After the initial pause, organisations were able to resume providing parenting programmes and other psychosocial support by changing the physical settings where programmes are delivered, reducing groups sizes, and using remote formats. These findings support the view that digital interventions can help reach more families (Calam et al., 2008). The pandemic has accelerated the adoption of remote delivery (Canário et al., 2022; Cluver et al., 2020; Lachman et al., in submission) and will perhaps be a critical juncture for wider use of digital technology in working with families in the future (Itzhaki-Braun, 2021). However, as our research highlights, there are barriers to remote delivery of family programmes in terms of sufficient access and relationship-building. Our findings highlight the importance of considering barriers to access when planning, service delivery, for instance through budgeting for and providing staff and families with mobile data and devices or identifying ways these can be accessed. We are also conducting a systematic review to further learn from how other parenting programmes were adapted to the pandemic (Fang et al., 2022).

Barriers to digital delivery have also been identified in other projects examining remotely delivered family programmes (Barnett et al., 2021; Canário et al., 2022; Franz et al., 2021). One study noted differences in adapting to remote delivery between settings in USA and South Africa. In South Africa, due to more limited computer and internet access, implementation required more extensive training of the facilitators in the digital version and creating a low-bandwidth and offline versions of the programme (Franz et al., 2021). Programmes that combine digital and in-person elements may help combine human connection with more flexible access to services.

As our findings show, the pandemic brought about new tasks and responsibilities for staff, and challenges associated with working from home as well as health risks from in-person work. This is consistent with findings from studies examining the experiences of community and social workers during the pandemic, which found resilience as well as fatigue (Ajibo, 2020; Banks et al., 2020; Gunhidzirai et al., 2022). Additional investment and attention are needed to examine ways to support frontline staff and organisations working with families and communities to reduce violence and promote positive parenting and wellbeing in the difficult and ever-changing circumstances of the pandemic and other crises.

### Strengths and limitations

A limitation of this study is that it was designed around two specific parenting programmes. We also focused on the experiences of service providers as we did not have the resources to collect data from families. The findings may also be skewed as over a third of the participants represented organisation A, the largest of the three community-based organisations taking part in the study. However, we have focused on the findings that reflect views from multiple organisations and contrasted differing responses, where relevant. We have also sought to maximise the internal validity of the study by conducting validation meetings with participants and asking for feedback on the findings at multiple times during the study. Another strength of our study is its representativeness across the three major languages in Cape Town – isiXhosa, Afrikaans and English. Where translations were employed during and after the interviews, the translation may have altered some of the content, but this was mitigated through working with interpreters and translators proficient in the relevant languages and conducting translation quality checks.

### Conclusions

This study provides insights on working with families in a low-resource setting during the first year of the COVID pandemic, which may generalise to other health and social programmes. Future research should explore whether responsive adaptations started during the pandemic continue to be used and how they evolve over time.

## Supplementary Material

Supplemental Material

Supplemental Material

## Data Availability

Data are not publicly available due to the ethical necessity of maintaining participant confidentiality. All data collection tools are available at https://osf.io/p6djt/?view_only=.
