# Supplementary material for: Adaptations and staff experiences in delivering parenting programmes and other family support services in three community-based organisations in Cape Town, South Africa during the COVID pandemic
Source: Glob Public Health. 2022 Nov 7;18(1):2129725. doi: 10.1080/17441692.2022.2129725 (PMC10802685; doi:10.1080/17441692.2022.2129725)
Supplement: Supplemental Material [file RGPH_A_2129725_SM1264.pdf]

[illegible]

(Interview the programme manager, coordinator, etc. as advised by the implementing partner.)

INTERVIEW SUMMARY	
Date	
Name of Interviewer	
Organisation	
Respondent Name	
Gender	
Role in the Organisation	
Programme Title	*Maybe at the start of the interview, or in advance by email, the interviewer can confirm details such as:*<br>- *PLH version implemented*<br>- *Project name and duration*

<u>Introduction to Interview</u>

I would like to thank you for taking the time to participate in this interview. I am from [INSERT ORGANISATION AFFILIATED WITH] and work as part of the Parenting for Lifelong Health Scale-Up of Parenting Evaluation Research study or PLH-SUPER for short. Through this study, we hope to learn more about how the programmes are implemented across different contexts.

This questionnaire is divided into two sections. Each section contains about ten questions. Section One relates to how COVID-19 has affected your work as a service provider and how you adapted or coped with the difficulties or changes during this period. In Section Two, we would like to learn about your knowledge and experience in the delivery of PLH programmes [USE LOCAL PROGRAMME NAME IF RELEVANT]. The full interview will take 60-90 minutes to complete. You have a choice on how you wish to proceed with the interview. For instance, you can take the interview all at once or decide to take a short break of about 15 minutes between sections 1 and 2. You can also decide to reschedule section 2 if you prefer.

There are no right or wrong answers or comments in this interview, and we are interested in having an open conversation on your views and suggestions. We will record the discussion on audio recorders to ensure that we capture your views accurately. We will also provide you with a recording of the interview of review and further input where necessary.

The discussion will be confidential and only viewed by the PLH-SUPER team. In published reports, we will keep your name and identity private. Your de-identified data may be shared with other researchers in the future.

If any of the questions asked are unclear, please let me know so that I can explain them differently. Your honest answers will be appreciated.

Before we begin, are there any questions?

SECTION 1: COVID-19 RELATED INTERVIEW QUESTIONS

A. Effects of COVID-19 on service delivery
COVID-19 has affected many individuals and services around the world, and we are interested to find out more about how it has affected your work and the work of your organisation, in terms of delivering PLH and other work that you do.

1. Is your organisation currently able to provide support for families? If yes, how?
2. Are you currently able to provide support for families in your work? If yes, how?
3. What has been your biggest professional challenge during the COVID-19 pandemic/social isolation?
4. If you knew a year ago that COVID-19 would happen, how would you prepare your organization to keep doing their work?'

B. Adaptation/coping approaches/resilience

5. Has your work on supporting families changed, If yes, how?
6. What has been helpful so far in continuing your work with families?
7. What resources or changes would be helpful for your work with families?

C. Parenting for Lifelong Health

8. Do you use anything from the PLH programme to help you with your work?
    - Prompts: If yes, what do you use? PLH tools and content? Ideas from PLH? Home visits?
    - Have you seen the PLH COVID tip-sheets? (If not, interviewer offers to share).
9. Is there anything the PLH programme could have done to help you during COVID in relation to your work?

We are thinking about conducting further research as the COVID-related restrictions continue, so we can find out about programme delivery without conducting in-person interviews or events. To help plan this, we would appreciate your advice on participating in research online or remotely.

D. Feasibility of online qualitative data collection [This will also be answered by the observations of the interviewer of the data collection process.]

10. What was your experience/How was it for you to participate in this online/phone interview? Is there anything you would recommend us to change?
11. Would you be willing to participate in research using text-based conversations? If so, which platform would you find most convenient? (WhatsApp, Facebook, Telegram, other?)

SECTION 2: QUESTIONS RELATING TO PLH/PARENTING PROGRAMME

Exploration and adoption

A. Usually, before organisations/government adopt evidence-based programmes, they explore the health needs of communities to identify the suitable evidence-based programmes to address those needs. I will ask you questions related to your decision to adopt PLH programmes.

1. Please tell me about the organisational history/journey to the adoption of PLH?

    Probes: ask only when necessary

- *Were there policies/procedures that guided your decision to adopt the PLH programme?*
- *Please tell us more about these policies*
- *Would you like to share these policy documents?*
- *Who else (organisation/agency) was involved in adopting the PLH programme/s?*
- *What was their role in the adoption of PLH?*
- *In your opinion, what is the overall goal of the PLH programme in your country?*

2. Did you encounter any challenges in the process of adopting the PLH programme?

   Probes: ask only when necessary
   - *If any, what were they?*
   - *How did they affect the adoption process?*
   - *What some possible solutions to these challenges?*

Preparation and adaptation

B. Before the delivery of evidence-based programmes, organisations usually develop an implementation plan for how best to deliver evidence-based programmes. The Implementation phase is then guided by the planned implementation supports from the preparation phase. I will now ask you questions related to the preparation and implementation of PLH programme delivery.

1. How did your organisation prepare for the rollout of the PLH programme?

   Probes: ask only when necessary
   - *How much time was allocated to preparing for PLH programme rollout? Do you think this time was enough, why?*
   - *Which other organisations were involved in preparing for PLH programme delivery, and why?*

2. What fidelity measures do you have in place to monitor or ensure that the intervention is delivered according to the manual or specifications?

   Probes: ask only when necessary
   - *What are some of the biggest challenges/threats to programme delivery?*
   - *What approaches do you use to overcome these barriers?*

3. Did you encounter any need to adapt or modify the PLH programme before or during delivery? *If so, what were these modifications and the reasons for them?*

C. Lessons from implementation:

1. What have you learnt from implementing the PLH programme?

   Probes:

   - *What works and what does not work?*
   - *What would you improve if you could?*

- *What were the challenges and barriers of implementation?*

How are you involved in monitoring and evaluating the programme delivery and impact? *(Only ask if involved in monitoring and evaluation, if not skip to Section D)*

Probes:

- *Which aspects of the programme has been monitored/evaluated?*
  - *family outcomes*
  - *family attendance and engagement*
  - *quality of delivery by facilitators and community service organisarions (CSOs) (if relevant)*
  - *effects on the facilitator skills, and the organisation*
- *If so, please describe the methods used to evaluate your programme and results from the evaluation (you can also refer to existing documents or publications).*
- *Please describe some of the challenges and barriers to monitoring the PLH programme.*

2. Do you think there is anything about your organisation that made it easier or harder to take up or deliver PLH?

   Probes:

   - *How, do you think, the structure of your organisation affected PLH implementation? What about the funding mechanism for your organisation?*
   - *How do you think the culture in your organisation, for instance around support for new initiatives, affected PLH implementation?*
   - *Do you think the leadership of the organisation affected PLH implementation?*
   - *Do you think the attitudes among staff affected PLH implementation?*

3. Can you tell me anything about programme costs and how these are recorded? *If not, is there someone else I can talk to about this? (Who?)*

D. Sustainment and scale-up

There is a growing need for the sustainment and scale-up of evidence-based programmes so that they reach large numbers of beneficiaries. I will now ask you questions related to the sustainment and scale-up of PLH programmes.

1. What measures do you have in place to ensure that the programme continues functioning for a long time?

   Probes:
   - *Is yes, what are those measures?*
   - *Please describe some of the challenges in maintaining the impact of the programme on beneficiaries.*
   - *What are some of the challenges in maintaining your programme within your organisation or agency?*

- *How is the programme integrated or institutionalised within an ongoing service delivery system? If it is not, what are some of the challenges?*

2. In your opinion, is the PLH programme ready to be scaled up?

Probes:

- *If not, what would you suggest is required before scale up?*
- *If so, how do you plan to scale up the PLH programme?*
- *What is the role of government in maintaining and scaling the programme?*
- *What may be barriers and facilitators to taking the programme to scale (i.e., financial, human, and organisational resources)?*
- *How would the private sector be involved in helping to support and maintain this programme at scale?*
