# Supplementary material for: Adaptations and staff experiences in delivering parenting programmes and other family support services in three community-based organisations in Cape Town, South Africa during the COVID pandemic
Source: Glob Public Health. 2022 Nov 7;18(1):2129725. doi: 10.1080/17441692.2022.2129725 (PMC10802685; doi:10.1080/17441692.2022.2129725)
Supplement: Supplemental Material [file RGPH_A_2129725_SM1262.pdf]

I would like to thank you all for taking the time to participate in this focus group discussion. I am from [INSERT ORGANISATION AFFILIATED WITH] and work as part of the Parenting for Lifelong Health Scale-Up of Parenting Evaluation Research study or PLH-SUPER for short. Through this study, we hope to learn more about how the programmes are implemented across different contexts.

This focus group discussion is divided into two sections. Each section contains about ten questions. Section One relates to how COVID-19 has affected your work as a service provider and how you adapted or coped with the difficulties or changes during this period. In Section Two, we would like to learn about your knowledge and experience in the delivery of PLH programmes [USE LOCAL PROGRAMME NAME IF RELEVANT]. The full interview will take 60-90 minutes to complete. You have a choice on how you wish to proceed with the interview. For instance, you can take the interview all at once or decide to take a short break of about 15 minutes between sections 1 and 2. You can also decide to reschedule section 2 if you prefer.

There are no right or wrong answers or comments in this interview, and we are interested in having an open conversation on your views and suggestions. We will record the discussion on audio recorders to ensure that we capture your views accurately. We will also provide you with a recording of the interview of review and further input where necessary.

All points made during the discussion will be confidential and only viewed by the PLH-SUPER team. In published reports, we will keep your name and identity private. Your de-identified data may be shared with other researchers in the future.

If any of the questions asked are unclear, please let me know so that I can explain them differently. Your honest answers will be appreciated.

Before we begin, are there any questions?

SECTION 1: COVID-19 RELATED INTERVIEW QUESTIONS

A. Effects of COVID-19 on service delivery

COVID-19 has affected many individuals and services around the world, and we are interested to find out more about how it has affected your work and the work of your organisation, in terms of delivering PLH and other work that you do.

1. Is your organisation currently able to provide support for families? If yes, how?
2. Are you currently able to provide support for families in your work? If yes, how?
3. What has been your biggest professional challenge during the COVID-19 pandemic/social isolation?
4. If you knew a year ago that COVID-19 would happen, how would you prepare your organization to keep doing their work?'

B. Adaptation/coping approaches/resilience

5. Has your work on supporting families changed, If yes, how?
6. What has been helpful so far in continuing your work with families?
7. What resources or changes would be helpful for your work with families?

C. Parenting for Lifelong Health

8. Do you use anything from the PLH programme to help you with your work?
    - Prompts: If yes, what do you use? PLH tools and content? Ideas from PLH? Home visits?
    - Have you seen the PLH COVID tip-sheets? (If not, interviewer offers to share).
9. Is there anything the PLH programme could have done to help you during COVID in relation to your work?

We are thinking about conducting further research as the COVID-related restrictions continue, so we can find out about programme delivery without conducting in-person interviews or events. To help plan this, we would appreciate your advice on participating in research online or remotely.

D. Feasibility of online qualitative data collection [This will also be answered by the observations of the interviewer of the data collection process.]

10. What was your experience/How was it for you to participate in this online/phone interview? Is there anything you would recommend us to change?
11. Would you be willing to participate in research using text-based conversations? If so, which platform would you find most convenient? (WhatsApp, Facebook, Telegram, other?)

SECTION 2: QUESTIONS RELATING TO PLH/PARENTING PROGRAMME

Overall experience

1. Can you please describe what your experience has been like delivering the PLH programme?
2. How were you able to balance your delivery of this programme with other work that you have?

    Probe:
    - *How is PLH related to other services you offer?*

3. In what ways has your experience in delivering the programme affected your views on how to work with parents on strengthening parent-child relationships?
4. How have participants benefitted from the programme?

A. Training and support

1. What are your overall views on the training that you received?
2. How well did the training prepare you to deliver the programme?
3. How well did the training prepare you to provide coaching to facilitators?
4. What are your thoughts on the coaching provided? How could these have been improved?
5. What are your views on the administrative support that you received during delivery of the programme*? How could this have been improved?*

Probe:

- *What else could be done to improve your work and performance?*

B. Barriers and enablers of programme delivery:

1. What are the main barriers and enablers for the quality delivery of the programme?
2. What modifications did you make to the training, implementation and ongoing support?
3. Is the PLH programme helpful, *If so, how?*

Probes:

- *What aspects of the programme do participants enjoy the most?*
- *What information is most helpful to parents/caregivers?*
- *What feedback do you get from participants (formal/informal)?*
- *Are participants able to implement the recommended behaviours at home?*
- *What are some of the challenges they have identified?*
- *How could the programme be improved to address some of these challenges?*
- *Do you think there is dissemination to other community members, of the ideas or practices taught in the programme?*
- *Has the programme benefited you as facilitators? If so how?*

C. Cultural acceptability

1. Please tell us about the cultural acceptability and suitability of the programme.

Probes:

- *How do you feel about the cultural acceptability of the programme?*
- *How do participants feel about the cultural acceptability of the programme?*
- *What are your thoughts on the potential for the provision of this parenting programme as a regular service? What challenges and benefits might there be?*
- *What other professionals or community members do you think may be appropriate to deliver this programme?*

D. Programme sustainability and scale-up

1. What would be necessary to make it possible for you and other facilitators/coaches to deliver the programme on a long-term basis?
2. What are the possible challenges towards the making this possible?
